# One-step detection of *Bean pod mottle virus* in soybean seeds by the reverse-transcription loop-mediated isothermal amplification

**DOI:** 10.1186/1743-422X-9-187

**Published:** 2012-09-07

**Authors:** Qi-Wei Wei, Cui Yu, Shu-Ya Zhang, Cui-Yun Yang, Karwitha Miriam, Wen-Na Zhang, Dao-Long Dou, Xiao-Rong Tao

**Affiliations:** 1 Key Laboratory of Integrated Management of Crop Diseases and Pests, Ministry of Education, Department of Plant Pathology, Nanjing Agricultural University, Nanjing 210095, People’s Republic of China; 2 Shanghai Entry Exit Inspection and Quarantine Bureau, Shanghai 200135, People’s Republic of China

**Keywords:** *Bean pod mottle virus* (BPMV), RT-LAMP, Rapid detection, Soybean seeds

## Abstract

**Background:**

*Bean pod mottle virus* (BPMV) is a wide-spread and destructive virus that causes huge economic losses in many countries every year. A sensitive, reliable and specific method for rapid surveillance is urgently needed to prevent further spread of BPMV.

**Methods:**

A degenerate reverse-transcription loop-mediated isothermal amplification (RT-LAMP) primer set was designed on the conserved region of BPMV CP gene. The reaction conditions of RT-LAMP were optimized and the feasibility, specificity and sensitivity of this method to detect BPMV were evaluated using the crude RNA rapidly extracted from soybean seeds.

**Results:**

The optimized RT-LAMP parameters including 6 mM MgCl_2_, 0.8 M betaine and temperature at 62.5-65°C could successfully amplify the ladder-like bands from BPMV infected soybean seeds. The amplification was very specific to BPMV that no cross-reaction was observed with other soybean viruses. Inclusion of a fluorescent dye makes it easily be detected in-tube by naked eye. The sensitivity of RT-LAMP assay is higher than the conventional RT-PCR under the conditions tested, and the conventional RT-PCR couldn’t be used for detection of BPMV using crude RNA extract from soybean seeds.

**Conclusion:**

A highly efficient and practical method was developed for the detection of BPMV in soybean seeds by the combination of rapid RNA extraction and RT-LAMP. This RT-LAMP method has great potential for rapid BPMV surveillance and will assist in preventing further spread of this devastating virus.

## Background

*Bean pod mottle virus* (BPMV) is a member of the genus *Comovirus* in the family *Comoviridae*. It is efficiently transmitted in nature by several vectors including beetle and mechanically via farm machinery within and between soybean fields. Currently, BPMV is widespread in world’s major soybean-growing areas. The deleterious effects of BPMV infection not only reduce soybean yield but also affect seed quality. In addition, it causes huge economic losses and sharpen the dissatisfaction from soybean growers and soybean marketers [1]. A sensitive, reliable and specific method for rapid surveillance is urgently needed to prevent further spread of BPMV.

There are several methods for BPMV surveillance, observation of viral symptoms, scanning of virus particle morphology and serological testing are among the early common practical methods for virus detection in the field. Currently, enzyme activity-linked immunosorbent assay (ELISA) [2], one-step IC-RT-PCR [3], reverse transcription-polymerase chain reaction (RT-PCR) [4] and semi-nested RT-PCR [5] are some frequently used methods for detecting viruses and assay. ELISA is a reliable method for detecting soybean viruses and it is suitable for high-throughput assay [6]. However, the sensitivity of ELISA may not be high enough to detect viruses in soybean seeds. RT-PCR assay is more sensitive but it needs to prepare pure RNA template and it requires an expensive thermo-cycle and is relatively time-consuming.

Loop-mediated isothermal amplification (LAMP) is an established nucleic acid amplification method offering rapid, accurate and cost-effective diagnosis of infectious diseases [7]. The LAMP assay employs two pairs of primers that recognize six regions of a target sequence, and amplifies specifically and isothermally the target sequence thus eliminating the need for thermo-cycling. Presently, RT-LAMP has been used to detect various viruses in diseased plants such as *Tomato spotted wilt virus *[8], *Plum pox virus *[9], *Peach latent mosaic viroid *[10] and viruses in rice [11].

In this study, we developed a rapid and feasible method for one-step detection of BPMV in soybean seed by the combination of rapid RNA extraction and RT-LAMP. Our results demonstrated that this RT-LAMP method has great potential for rapid BPMV surveillance and will assist in preventing further spread of this BPMV.

## Results

### Design of the RT-LAMP primer set for BPMV

**Figure 1:**
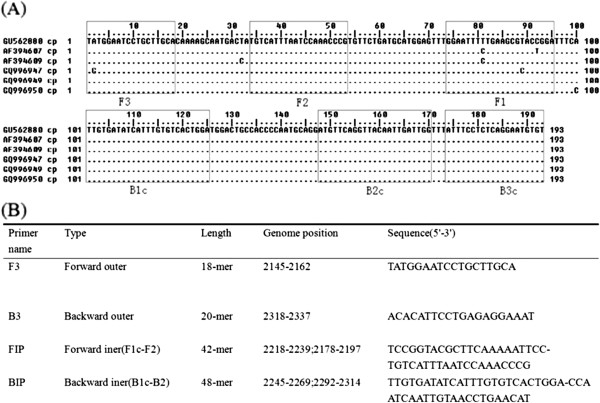
**Alignment of the selected region of BPMV CPs and the RT-LAMP primer set.** (**A**) Alignment of the selected region of BPMV CP used for designing the RT-LAMP primer. The isolate or strain of BPMV used in the alignment are as follows: isolate IA-Di1 (GU562880.1), strain K-Hancock 1 (AF394607.1), strain K-Hopkins 1 (AF394609.1), isolate Iowa-Prignitz 10 (GQ996947.1), isolate Iowa-Desmodium illinoense 1 (GQ996949.1 and GQ996950.1). (**B**) The primer set selected for RT-LAMP. BPMV isolate IA-Di1 were used as reference sequence.

In order to design a degenerate BPMV RT-LAMP primer set to amplify different BPMV isolates, five primer sets were initially generated in Primer Explorer V4 software using coat protein (CP) gene of BPMV as target. Nucleotide sequences of CP from different BPMV isolates were retrieved from GenBank and they were aligned to analyze the highly conserved regions in the CP gene [12,13]. After a series of careful manual analyzes, one set of primer on the most conserved regions of BPMV CP gene sequence was selected for further evaluation (Figure 1).

**Figure 2:**
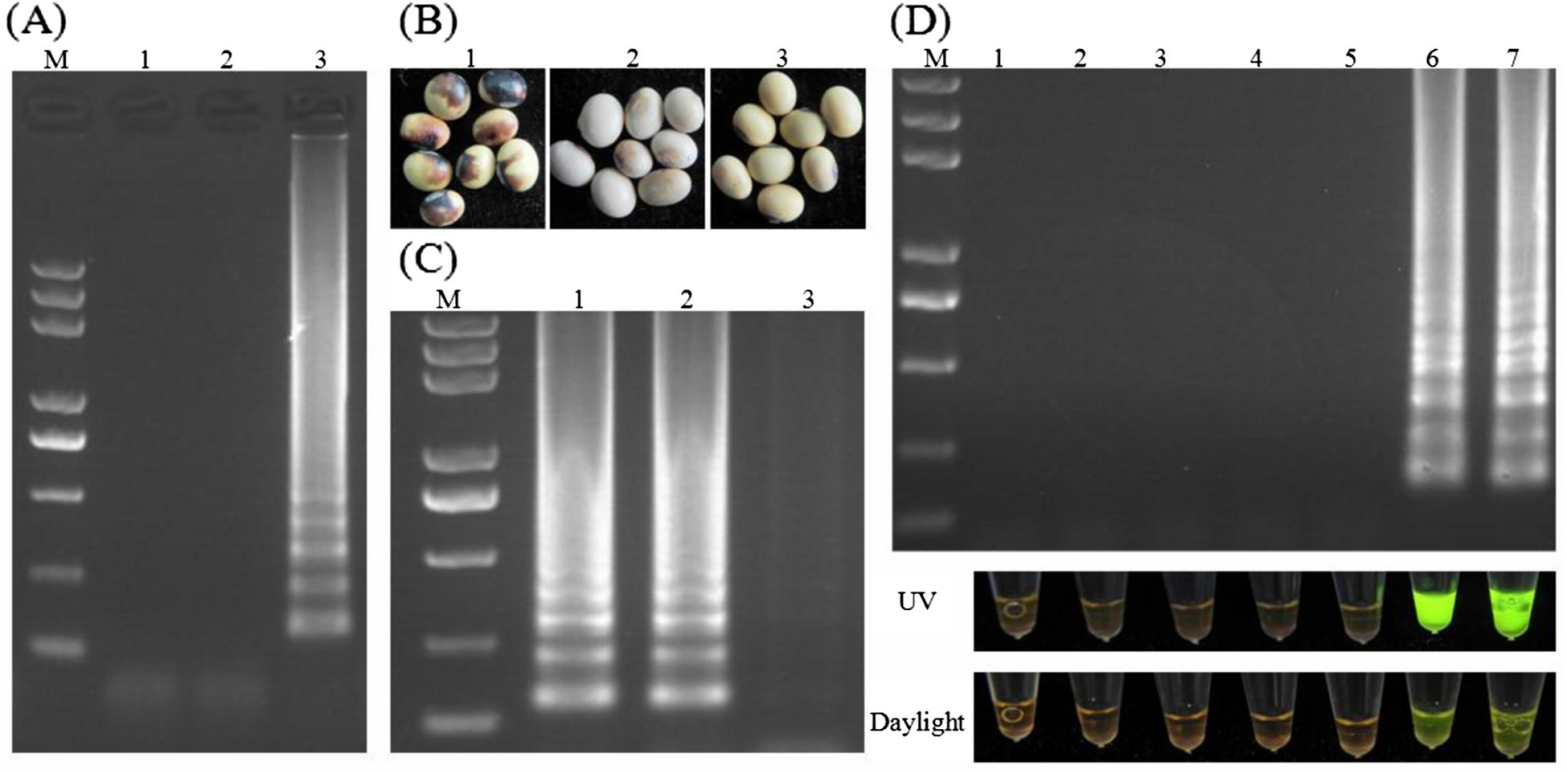
**RT-LAMP detection of BPMV in soybean seeds.** (**A**) The functional test of RT-LAMP primer set by LAMP assay. 1: Negative control without DNA template; 2: Empty plasmid; 3: Plasmid harboring BPMV CP. (**B**) Soybean seeds infected by BPMV and healthy soybean seeds. 1: Sample 1; 2: Sample 2; 3: Healthy soybean seeds. (**C**) One-step RT-LAMP was tried to detect BPMV in soybean seeds. 1: BPMV sample 1; 2: BPMV sample 2; 3: Healthy soybean seeds. (**D**) Specificity analysis of the RT-LAMP assay for BPMV in soybean seeds. Amplified products were observed by electrophoresis (upper panel) and visually detected by adding SYBER GREEN I for color changes (lower panel). 1: Healthy soybean seed; 2 and 3: Soybean seeds harboring SMV; 4 and 5: Soybean seeds harboring CMV; 6 and 7: Soybean seeds harboring BPMV (sample 1 and 2). 3 μL of (**A**), (**C**) and (**D**) amplified products were checked on a 1% agarose gel electrophoresis in TBE. M: 2 kb DNA marker.

To determine whether the RT-LAMP primer set can be used for detection of BPMV, a plasmid containing BPMV CP gene was tested by LAMP assay using the reaction parameters: 20 mM Tris–HCl (pH 8.8), 10 mM KCl, 10 mM (NH_4_)_2_SO_4_, 8 mM MgSO_4_, 0.1% Tween 20, 0.8 M betaine, 0.2 μM each of two outer primers, and 1.6 μM each of two inner primers, 1.4 mM dNTPs, 8 units of Bst DNA polymerase, and 1.5 μL of the DNA template in 25 μL total reaction mixture,described in Le et al.*,* 2010 [14]. As shown by Figure 2A, the LAMP products of the positive plasmid harboring the genomic cDNA of BPMV CP gave a ladder-like band on the agarose gel. Whereas, the plasmid without the BPMV CP or negative control without DNA template did not generate the ladder-like band, indicating that the selected RT-LAMP primer set was adequate.

### Establishment of one-step detection of BPMV in soybean seed by combination of a rapid RNA extraction and the RT-LAMP

In order to establish a one-step detection of BPMV in soybean seed, crude RNA was extracted from ground soybean seeds in NaOH and immediately neutralized by Tris–Cl buffer. The crude RNA was assayed for RT-LAMP including AMV reverse transcriptase (0.05 U/μL in final volume) using the parameters described in Le et al. 2010 [14]. Unfortunately, we failed to amplify the ladder-like band from the BPMV infected soybean seeds (data not shown). We therefore made some adjustments on the amount of Mg^2+^, dNTP, outer primers and inner primers for the parameters of the RT-LAMP assay according to previous RT-LAMP studies [15-17], and the outcome of our RT-LAMP parameters are listed in Table 1. We demonstrated that these adjusted parameters could successfully amplify the ladder-like bands for the soybean seeds infected by BPMV (sample 1 and sample 2) in combination of rapid RNA extraction and the RT-LAMP assay (Figure 2C). Meanwhile, no band was amplified from healthy soybean seeds.

**Table 1:** Parameters of the RT-LAMP reaction

**Component (concentration) (supplier)**	**Volume (** μ**L) per 10** μ**L reaction**
Autoclaved PCR-grade MilliQ™water	2.8
10 × ThermoPol reaction buffer (containing 2 mM MgCl_2_) (New England Biolabs)	1
^*^25 mM MgCl_2_ (4 mM final) (TaKaRa)	1.6
10 mM dNTP’s (0.8 mM final) (TaKaRa)	0.8
FIP 20 μM (0.8 μM final) (Invitrogen)	0.4
BIP 20 μM (0.8 μM final) (Invitrogen)	0.4
F3 10 μM (0.2 μM final) (Invitrogen)	0.2
B3 10 μM (0.2 μM final) (Invitrogen)	0.2
5 M Betain (0.8 M final) (Sigma)	1.6
Bst DNA polymerase 8 U/μL (0.32 U/μL final) (New England Biolabs)	0.4
AMV reverse transcriptase 10 U/μL (0.05 U/μL final) (Promega)	0.05
DNA/RNA template or Crude RNA extract	0.6

*Total [MgCl_2_] is 6 mM, as the reaction buffer contains an addtional 2 mM.

### Specificity of BPMV detection by RT-LAMP

After the establishment of one-step detection of BPMV in soybean seed, the soybean seeds containing *Soybean mosaic virus* (SMV) or *Cucumber mosaic virus* (CMV) certified by conventional RT-PCR previously were tested by RT-LAMP to determine whether the RT-LAMP amplification was specific to BPMV. As shown by Figure 2D (upper panel), the RT-LAMP amplification of the soybean seeds infected by BPMV (sample 1 and sample 2) could generate ladder-like bands on the agarose gel. While the soybean seeds containing SMV or CMV did not show the similar band as well as the negative control, indicating that RT-LAMP is specific to BPMV.

### In-tube detection of RT-LAMP by naked eyes

RT-LAMP products gave a ladder-like band on the agarose gel but we found that the positive results could not be visually observed in-tube by naked eyes determined by turbidity or the white precipitate of magnesium pyrophosphate in the LAMP reaction. Therefore, a drop of SYBER GREEN I was used to help the observation of results after the reaction was completed. As shown by Figure 2D (lower panel), the color of the RT-LAMP reaction containing soybean seed sample 1 or sample 2 changed from orange to green, which was easily visible to naked eyes under the UV light or daylight. On the other hand, there was no color change in the reaction tubes of other soybean seeds without BPMV.

### Optimization of reaction temperature in one-step RT-LAMP

**Figure 3:**
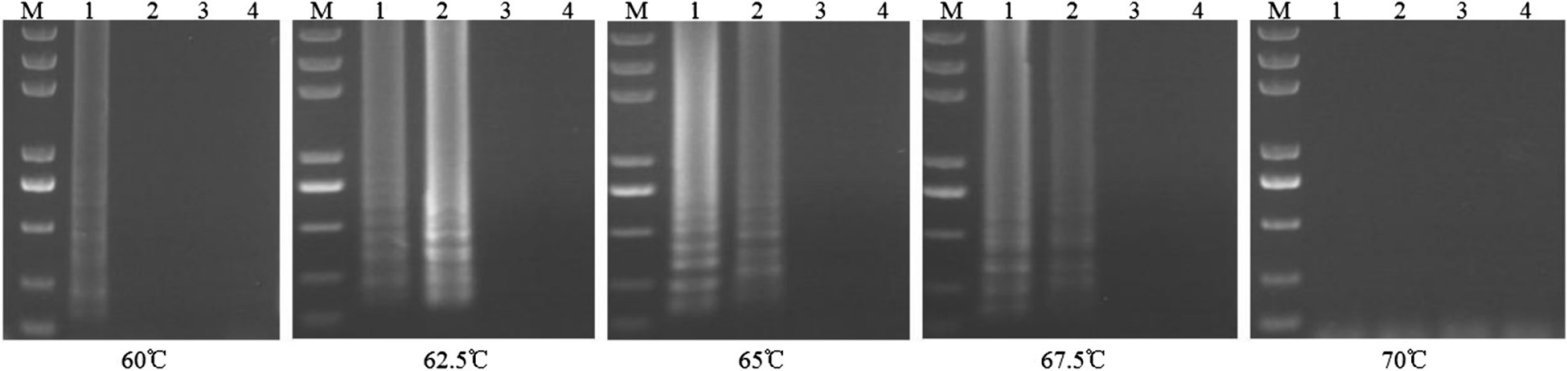
**Optimization of reaction temperature of the RT-LAMP for the detection of BPMV in soybean seeds.** RT-LAMP was carried out at 60, 62.5, 65, 67.5 and 70°C to detect BPMV in soybean seeds. 3 μL of amplified products were observed on a 1% agarose gel electrophoresis. M: 2 kb DNA marker; 1 and 2: Soybean seeds harboring BPMV (sample 1 and 2): 3: Healthy soybean seeds; 4: Negative control without DNA template.

In the preliminary detection of BPMV in soybean seeds using crude RNA extract, we set up 60°C and 65°C for RT-LAMP reaction temperature according to earlier studies[18-20], but we found that the reaction at 60°C was always not consistent in our experiments. In order to select an optimum amplification temperature for RT-LAMP reactions, the different temperature ranging from 60°C-70°C were assayed for RT-LAMP. In Figure 3, the ladder-like bands on the gel were successfully detected at 62.5 and 65°C when using crude RNA extract from soybean seed, the band was a little weaker at 67.5°C but it was absent at 70°C. Occasionally the band occurred at 60°C. These data suggest that 62.5-65°C is an optimum amplification temperature for one-step RT-LAMP.

### Sensitivity of the RT-LAMP vs conventional RT-PCR

**Figure 4:**
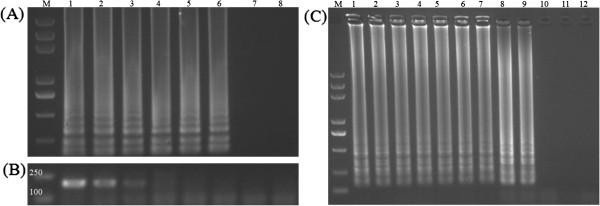
**Sensitivity of the RT-LAMP vs conventional RT-PCR.** (**A**) Serial 10-fold dilutions of total RNA of BPMV soybean seeds sample 1 extracted using Trizol Reagent were subjected to RT-LAMP from 100 to 0.0001 ng. 1–7: 100, 10, 1, 0.1, 0.01, 0.001, 0.0001 ng RNA was used as template, respectively; 8: No RNA template. (**B**) Total RNA same as (A) was used to one-step RT-PCR. (C) Crude RNA extract of sample 1 were used as templates in RT-LAMP from 0.2 to 2.0 μL at intervals of 0.2 μL. 1–10: 0.2, 0.4, 0.6, 0.8, 1.0, 1.2, 1.4, 1.6, 1.8, 2.0 μL RNA was used as template, respectively; 11: Healthy soybean seeds; 12: No RNA template. 3 μL of amplified products were checked on a 1% agarose gel electrophoresis. M: 2 kb DNA marker.

To characterize the sensitivity of RT-LAMP detection of BPMV in soybean seeds compared to conventional RT-PCR, total RNA extracted from soybean seed sample 1 using Trizol was subjected to RT-LAMP and one-step RT-PCR. The total RNA was serially diluted 10-fold from 100 to 0.0001 ng that had been quantified by spectrophotometer. As shown in Figure 4 A & 4B, RT-LAMP successfully detected as little as 0.001 ng RNA, while the detection limit of RT-PCR was 1 ng RNA. Comparing RT-LAMP and RT-PCR assays revealed that the sensitivity of RT-LAMP was 100 to 1000 times higher than the conventional RT-PCR under the conditions tested as indicated by the presence of a 193 bp amplicon.

Interestingly, we found that one-step RT-PCR could not amplify any band using crude RNA extract as template whereas ladder like bands were easily detected in RT-LAMP reaction by using same crude RNA extract. Nevertheless, we couldn’t quantify the exact amount of RNA using crude extract in each reaction. To determine an optimum amount of crude RNA extract that should be included in each reaction, ten various volumes of template ranging from 0.2 to 2 μL at intervals of 0.2 μL were further tested in a 10 μL total volume of one-step RT-LAMP reaction. Figure 4C showed the specific ladder-like band of RT-LAMP could be amplified by using 0.2, 0.4, 0.6, 0.8, 1.0, 1.2, 1.4, 1.6 and 1.8 μL of template, respectively. No band was amplified by using 2.0 μL of template in 10 μL reaction mixture. This result suggest that a quite broad range volume of the crude template can be added into RT-LAMP reaction, but too much crude template may interfere with the reaction, so it should be avoided.

## Discussions

In this study, a highly efficient and practical method for the detection of BPMV in infected soybean seed was developed by the combination of rapid RNA extraction and RT-LAMP. For the design of the RT-LAMP primer set, it is important to select the most conserved regions of the nucleotide sequence for each virus. Subsequently, nucleotide sequence analysis of several strains/isolates containing two distinct subgroups of BPMV strains [12,13] were used and proved the specificity and degeneracy of the primers. The amplified products of BMPV from crude RNA extract of soybean seeds could be easily detected as a ladder-like pattern by agarose gel electrophoresis or visually observed under UV light or daylight by adding fluorescent dye. The positive results could not be easily visualized by judgment of white precipitation in our system. Possibly, because the insoluble magnesium pyrophosphate does not accumulate high enough in the system using crude RNA extract or the BPMV concentration in soybean seeds is very low so that it cannot generate high amount of magnesium pyrophosphate [21].

The optimum temperature range of the RT-LAMP for crude RNA extract from soybean seeds ranged from 62.5–65°C, too high temperature might cause the inactivation of the enzyme or the instability of reaction that could affect the stability, sensitivity and strength of the detection [10]. While many studies use 60°C for the RT-LAMP amplification, this temperature did not work consistently in our system. It could be that some unknown factors in the crude RNA extract may have interfered with the RT-LAMP reaction at this temperature. RT-LAMP could detect BPMV from total RNA diluted up to 0.001 ng, compared to 1 ng RNA detected by convetional RT-PCR, suggesting that RT-LAMP has 100 to 1000 times more sensitive than the conventional one-step RT-PCR under the conditions tested [10,14,22,23]. When the total RNA purified using Trizol was replaced with the rapid RNA extract as template for RT-LAMP and RT-PCR, the RT-LAMP could amplify ladder-like bands when the volume of template was increased from 0.2-1.8 μL, but the conventional RT-PCR was not able to. It may be due to the fact that high levels of rapid RNA extract destructed the system of RT-PCR reaction or the low-dose of rapid RNA extract beyond the detection limit of RT-PCR. Consequently, it seems that being able to use rapid RNA extract as template for amplification was a dominant feature of RT-LAMP and a key factor of this method to shorten the time of BPMV detection [14].

## Conclusions

One-step RT-LAMP is a rapid, efficient, sensitive and highly specific method for the detection of BPMV in infected soybean seed by combining rapid RNA extraction with RT-LAMP. These predominant features of this method make it be potentially useful for rapid BPMV surveillance and assistant in preventing further spread of BPMV.

## Methods

### Source of materials

In this study, soybean seeds containing BPMV were provided by the Shanghai Inspection and Quarantine Bureau, China. Soybean seeds containing SMV and CMV were stocks in our laboratory. Their evidence of containing BPMV, SMV, CMV or no virus had been pre-confirmed by conventional RT-PCR. The BPMV CP in the positive plasmid was amplified from soybean by RT-PCR, and it was inserted into pMD_19_-T Vector (TakaRa, Dalian, P. R. China) and sequence confirmed.

### Sequence analysis and primer design

Several BPMV strains/isolates containing two distinct subgroups were obtained from GenBank and completed multiple alignment analysis by BioEdit 7.0.1 software. The conserved region in the coat protein coding region of BPMV was selected for primer design. Five sets of primers were initially designed using BPMV CP as target by Primer Explorer V4 software each included two outer primers (F3 and B3) and two inner primers (FIP and BIP). After a series of careful manual analysis, one set of those primers in the C-terminus of the coat protein was finally selected for further evaluation. This definitive RT-LAMP primer was designed within nucleotide positions 2145–2337 of the BPMV isolate IA-Di1 (Accession No. GU562880) used as the reference sequence. Primer details are listed in Figure 1 and all relative sequence data were retrieved from GenBank.

### RNA extraction

Total RNA used in sensitivity comparison of the RT-LAMP and conventional RT-PCR was extracted using Trizol Reagent (Invitrogen, Madison, USA) according to manufacturer’s instructions. RNA was dissolved in DEPC-treated water, and stored at −70°C before use. The method of rapid RNA extraction was as follow: 100 mg of soybean seed powder was mixed in 400 μL of 0.5 M NaOH and kept shaking 1–2 min. 10 μL of the resulting solution was diluted quickly with 490 μL of 100 mM Tris–Cl buffer (pH 8) and 1.5 μL of the final solution was used as template in 25 μL RT-LAMP reaction [14].

### RT-LAMP reaction

The parameters of RT-LAMP reaction used in this study are listed in Table 1. RT-LAMP reaction was carried out at 65°C for 60 min; heating at 80°C for 5 min terminated the reaction. 3 μL of the RT-LAMP products were electrophoresed on 1% agarose gel stained with ethidium bromide or visually detected under the UV light or daylight by adding 0.3 μL of SYBER GREEN I (Invitrogen, Madison, USA) after the 10 μL reaction was completed. The best way to add SYBER GREEN I is dropping it on the inner surface of the lid of each reaction tube, after the reaction is completed, well mixing the fluorescent dye with the final reaction product.

### RT-PCR reaction

The one-step RT-PCR reaction system in a 10 μL reaction were mixed with the following reagents: 1μL 5 × MMLV buffer (Promega, Shanghai, P. R. China), 0.5 μL 10 × Taq plus PCR buffer (TaKaRa, Dalian, P. R. China), 0.3 μL 25 mM MgCl2 (TaKaRa, Dalian, P. R. China), 0.2 μL 10 mM dNTP (TaKaRa, Dalian, P. R. China), 0.2 μL 20 μM upstream primer (Invitrogen), 0.2μL 20 μM downstream primers, 0.1 μL 200 U/μL MMLV reverse transcriptase (Promega), 0.1 μL 5 U/μL Taq plus polymerase (TaKaRa, Dalian, P. R. China), 0.1 μL 200 U/μL RNAse Inhibitor (RRI) (TaKaRa, Dalian, P. R. China), 0.6 μL RNA template and add DEPC-treated water up to 10 μL. RT-PCR was carried out with a first reaction at 42°C for 30 min, then denatured at 94°C for 5 min and followed by 35 cycles of 94°C for 30 sec, 50°C for 30 sec and 72°C for 30 sec, and further extended by 72°C for 7 min. 3 μL of the RT-PCR products were electrophoresed on 1% agarose gel stained with ethidium bromide [22].

### Specificity analysis of the RT-LAMP to BPMV

The crude RNA extracts of soybean seeds infected by SMV, CMV or BPMV (sample 1 and sample 2) and healthy soybean seed were used as template in RT-LAMP. Each template was 0.6 μL in 10 μL reaction. The reactions were carried out at 65°C for 60 min; heating at 80°C for 5 min to terminate the reaction. 3 μL of reaction products were electrophoresed on 1% agarose gel in TBE stained with ethidium bromide or was detected by naked eyes under the UV light or daylight with extra-additive SYBER GREEN I.

### Optimization of reaction temperature

RT-LAMP was amplified at five different temperatures (60, 62.5, 65, 67.5 and 70°C). All reactions were amplified for 60 min then heating at 80°C for 5 min to terminate the reaction. The crude RNA extract of soybean seeds infected by BPMV (sample 1 and sample 2) and healthy soybean seed were used as template in RT-LAMP. Each template was 0.6 μL in 10 μL reaction. 3 μL of reaction products were electrophoresed on 1% agarose gel in TBE stained with ethidium bromide.

### Sensitivity comparison

To compare the difference of sensitivity between RT-LAMP and RT-PCR, total RNA extracted from BPMV infected soybean seed sample 1 using Trizol reagent were serially diluted 10-fold from 100 to 0.0001 ng and used as template in RT-LAMP or RT-PCR reactions, and each template was 0.6 μL. When determining the optimum amount of crude RNA extract as template in RT-LAMP, the crude RNA extract of infected soybean seed sample 1 was used in RT-LAMP from 0.2 μL to 2 μL at intervals of 0.2 μL. The reaction volume was 10 μL and they were carried out at 65°C for 60 min; heating at 80°C for 5 min to terminate the reaction. 3 μL of the reaction products were electrophoresed on 1% agarose gel in TBE stained with ethidium bromide.

## Abbreviations

RT: Reverse-transcription; IC: Interchange; UV: Ultraviolet; CP: Coat protein; PCR: Polymerase chain reaction; SMV: *Soybean mosaic virus*; CMV: *Cucumber mosaic virus*; BPMV: *Bean pod mottle virus*; RT-LAMP: Reverse-transcription loop-mediated isothermal amplification; ELISA: Enzyme activity-linked immunosorbent assay; DEPC: Diethyl pyrocarbonate.

## Competing interests

The authors declare that they have no competing interests.

## Authors’ contributions

QWW, XRT, DLD designed this study; QWW, CY, SYZ, CYY, WNZ performed lab work; QWW participated in data analysis and drafted the manuscript; XRT, DLD, KM critically reviewed the manuscript. All authors' have read and approved the final manuscript.
